# On the Medical Treatment of Cataract

**Published:** 1848-10

**Authors:** Wm. G. Smith

**Affiliations:** Great Falls, N. H.


### BUFFALO MEDICAL JOURNAL

AND

### MONTHLY REVIEW.

VOL. 4. OCTOBER, 1848. NO. 5.

### ORIGINAL COMMUNICATIONS.

ART. I.—*On the Medical Treatment of Cataract.*—By WM. G. SMITH, M. D. Communicated by letter to DR. JAMES BRYAN, M. D., to the College of Physicians and Surgeons of Philadelphia, Pa.

[We invite the attention of our readers to the following letter of Dr. Smith, communicated by James Bryan, M. D., President of the College of Physicians and Surgeons, and Chairman of Committee on Surgery. Dr. Bryan has published several cases of Cataract successfully treated upon the plan laid down in the following communication, and we understand that he has several now under treatment, which promise the same success as have attended his published cases.—ED. BUF. MED. JOUR.]

*To the President, Officers and Members of the College of Physicians and Surgeons of Philadelphia:—*

In the month of April, 1848, I sent a letter to Dr. James Bryan, your President, in which I gave an account of the medical treatment which I adopted in a case of cataract, which, together with several other cases of cataract cured by Dr. Bryan, and remarks on the same by him, have been published in Dr. Houston's valuable Medical Journal (July number, 1848,) Having since had two other cases of cataract, which I have cured by medical means, I think it my duty to report them, to be read at one of your meetings.

When I say that I have cured two cases of cataract, I do not wish it to be understood that the patients had entirely lost their sight. You will perceive by reading the cases, that they could see a little when placed under my care.

I am aware that the editor of a medical Journal in your city has said, that my curing a case of cataract was "all humbug." I hope it has not come to this, that a man must be put down because he has done what some other men supposed impracticable; if it be so, slow will be the advancement of medical science. This is a time of free thought, and it is the duty of every medical man to think and act for himself, and not be trammeled by any man, or body of men. But I have said enough on this point.

*Case 1st.*—*May 4th*, '48.—Mrs. D., aged 23, of delicate appearance, called on me to inquire if I could do any thing to help her sight. She said she had been a hard working woman, had taken care of a large number of boarders, for a long time, doing the washing, ironing and cooking for the whole of them, in consequence of which her eyes had been much exposed to the blaze and heat of a large fire. She could not distinguish her own children from others, even when in the same room, unless they were close at hand; she could see best in cloudy days, at the close of day, and in the morning when she first waked up; every thing she saw appeared to be in a cloud. She complained of pain in the head and eyes. Tongue was coated, bowels costive, no desire for food of any kind. By looking into the eyes I saw (instead of that black appearance which we see in the pupil of a healthy eye,) a dirty appearance, being more observable in the centre of the pupil than at the circumference; the lens of the left eye being more opaque than that of the right.

I then told the woman and her husband who was present, that the disease was cataract, and that there might be a chance for improved vision. They then told me that they had called on a physician of this place, who, after examining her eyes, said that the disease was cataract. I then directed her to wear a pair of gray glasses during the day, and remain quiet in a room moderately dark; to live on a farinaceous diet, with half a pint of milk daily.

*May 5th.*—Applied four large foreign leeches to the external angle of the left eye, and three to the external angle of the right, applied a blister behind each ear, and at night gave her six grains of blue mass, followed, the next morning, with two compound cathartic pills, which operated well.

*May 6th.*—The pain in the head and eyes is diminished, blisters have drawn well. Still she has no appetite. Gave her the following powder three times a day until the 17th instant, when the mouth became sore:—hydrarg. cum cret. grs.ii, sup. carb. soda iv grains, and sulp. quinine grs. ss.

*May 7th.*—Feels some better. Applied five leeches to external angle of right eye, and three to external angle of left eye, which bled freely.

*May 8th.*—Eyes and head feel much better, and can see better, bowels regular, tongue clean, and appetite improving; blisters behind the ears have healed; I now put one on the back of the neck, which drew well.

*May 9th.*—Patient is comfortable. Can now distinguish her children from others in any part of a light room. She continued her medicine, kept the blister discharging until the 17th, when I suspended the mercury and continued soda and quinine, and, in addition, gave two grains iodide of Potassium three times a day. I this day, May 17th, applied three leeches to the external angle of each eye, and placed a blister behind each ear. I continued her present medicine until the 29th May, occasionally moving the bowels by sulp. magnesia and senna, and keeping a free discharge from the blisters which I applied, alternately, behind the ears, and to the back of the neck.

*May 30th.*—I called on her; said she could see well, and also felt well. I then discontinued all treatment. Before I left the house I saw her pick up a small cambric needle from the floor without the aid of glasses. This day she had read a letter and wrote an answer to the same. She now, July 28th, does the work for her family, consisting of ten persons.

*Case 2d.—May 9th.*—Mrs. H., aged 50, called on me, complaining of pain in her head and eyes; said she could *see* but very little, and remarked that objects appeared to be in a mist. She has been subject to rheumatic pains in her limbs and head, with severe pain at the same time in her eyes. Her uncle has been blind, with cataract in both eyes, for the last fifteen years. She says she is afraid she shall be blind from the same disease, because, for several years past, she has suffered with the same symptoms as did her uncle for several years previous to his loss of sight.

Present state:—pulse strong and full; plethoric state of the system; bowels costive, tongue little coated, moderate appetite; pain in the limbs, joints, head, and eyes; cannot see any better than Mrs. D. The eyes presented the same change as those of Mrs. D., and the lens of each eye as much altered in color, the left one being the worst. As she could not remain in the place, but must return home, I advised her to send for her physician as soon as convenient, and request him to bleed her thirty ounces from her arm, which she did, and also to apply 12 American, or 6 foreign leeches in the immediate vicinity of each eye, and a blister behind each ear, directed her to take an infusion of salts and senna, with ten drops of the wine of colchicum seeds, every other morning, so as to procure three evacuations during the day, and to take 6 grs. of blue mass every night; to live on bread and water, keep in a moderately dark room during the day, and wear gray glasses. She came again to see me on the 19th of May, and reported herself much better, had not applied the leeches, being unable to procure any in her town, but sight was improved a little. I immediately applied 4 large Spanish leeches to the external angle of each eye, the bites of which bled freely for 8 hours. She then becoming faint, I was obliged to arrest the bleeding. The next morning she left for home, with instructions to take the medicine as before, the mercury to be suspended when the mouth became sore, and to live on bread and water. *May* 28—sent me word that the mouth was a little sore, had discontinued the mercury; and that all of her bad feelings and pains had left her, and her sight was rapidly improving—sent her word to take only the salts and senna every third morning, keep blisters discharging, and to live on a more nourishing diet. *June* 12—this woman called on me, and reported herself well. I then examined her eyes, and found that the opacity was entirely removed. Last week I saw this woman's relations, who informed me, that she could see as well as when I last saw her.

I believe any medical man, after reading the above cases, and those published by Dr. Bryan, will come to the conclusion, that there are some forms of cataract which may be cured in their early stages, at least, without an operation. This being the fact, I think it is the duty of every medical man, when a case of cataract is brought to him, to do all that he can to arrest, or remove it by constitutional and local means, and not say 'I can do nothing, you must wait till the cataract is matured, and, then run the risk of an operation.'

All writers on diseases of the eye, concur in the opinion that cataracts may be produced by congestion, common, or rheumatic inflammation of the different coats, or internal structure of the eye, their formation being attended in those cases with all the symptoms indicating an unusual determination of blood to the head, and frequently a general fulness of the system. This being the fact, why cannot those cases of cataract be removed in their early, or immature state, by alteratives, counter irritation, and antiphlogistics. ?

Again, the eye contains specimens of all the animal tissues, and, of course, the same morbid changes take place in it, in consequence of inflammation, as in other parts of the body, and are as frequently removed by the same remedies. When we have inflammation of the pleura, we have the secretion lessened, but, most frequently there is an effusion of serum, and, sometimes, an excess of the nutritive secretion appears on the exterior of the membrane in various forms, and the lung may become secondarily affected. Now inflammation produces the same morbid changes in the crystaline capsule, (which is a "sero-vascular membrane," and which "contains lymphatics on its inner or serous portion") in consequence of which we may have capsular cataracts, and if the lens becomes secondarily diseased, we may have capsulo-lenticular cataract. All physicians know that bleeding, purgatives, calomel, &c., will cure disease of the pleura, and regulate its secretions; and why will not the same remedies remove the same morbid changes in the crystaline capsule and lens, if resorted to before the absorbing properties of the capsule are much altered by disease? Sir David Brewster regards cataract, "as a disease which arises from the unhealthy secretion of the aqueous humor," and thinks that cataract may be cured in its early stages, and in proof of this, he adduces the case of his own eye, in which the disease had made considerable progress, and was cured by paying the greatest attention to diet and regimen, and abstaining from reading at night, and all exposure of the eyes to fatigue or strong lights. (You will see his views on this subject fully explained on the 626 & 7th pages of Lawrence on the eye, by Hays, published 1847.)

Allow me to say, in conclusion, that the treatment of cataract by medical means, I learned from Dr. James Bryan, whilst attending his valuable lectures on surgery, during the last winter.

Very respectfully yours,

WILLIAM G. SMITH.

GREAT FALLS, N. H., July 31st, 1848.

